# Potential distribution of endemic lizards from Brazilian restingas: The present announcing the end

**DOI:** 10.1002/ece3.11618

**Published:** 2024-11-20

**Authors:** Hugo Andrade, Luisa Maria Diele‐Viegas Costa Silva, Carlos Frederico Duarte Rocha, Antônio Jorge Suzart Argôlo, Eduardo José dos Reis Dias

**Affiliations:** ^1^ Laboratório de Biologia e Ecologia de Vertebrados (LABEV), Departamento de Biociências Universidade Federal de Sergipe – UFS Itabaiana Sergipe Brazil; ^2^ Laboratório de (Bio)Diversidade no Antropoceno Instituto de Biologia, Universidade Federal da Bahia Salvador Bahia Brazil; ^3^ Departamento de Ciências Biológicas Universidade Estadual de Santa Cruz Ilhéus Bahia Brazil; ^4^ Laboratório de Ecologia de Vertebrados, Departamento de Ecologia, Instituto de Biologia Roberto de Alcantara Gomes, Universidade do Estado do Rio de Janeiro ‐ UERJ Rio de Janeiro Brazil; ^5^ Programa de Pós‐Graduação Em Ciências Naturais na Área de Biodiversidade e Meio Ambiente Universidade Federal de Sergipe Itabaiana Sergipe Brazil

**Keywords:** conservation, endemic lizards, environmental heterogeneity, geographic barriers, restingas, threatened species

## Abstract

The restinga habitats are coastal psammophilous environments, with only 0.47% of the original area remaining in Brazil. This environment embraces at least 36 known species of lizards, 7 of them being endemic. Besides direct anthropogenic impacts, climate change raises new cautions on Brazilian restingas‐endemic lizards conservation. We evaluated the current and future potential distribution of the endemic lizards from Brazilian *restingas*, considering different climate change scenarios. We hypothesized shifts in the potential distribution of the restinga‐endemic lizards. We conducted ecological niche modeling to predict the potential distribution of Brazilian restingas‐endemic lizards. Here, we used an ensemble of three modeling algorithms (Bioclim, GLM, and SVM). In predicting the effects of climate change on their future distributions, we used intermediate and pessimistic socio‐economic pathway scenarios (SSP3 70 and SSP5 85, respectively) considering projections for 2081–2100. Furthermore, we calculate the extent of future potential distribution covered by the current spatial configuration of integral protection areas to assess if they will still be effective in conserving the species in the future. We did this by binarizing predicted potential distribution with a threshold of 0.8. Our data pointed out that the species will have their potential distribution area altered for 2081–2100, considering climate change scenarios. *Tropidurus hygomi*, *Glaucomastix abaetensis*, and *G. itabaianensis* overlapped along their predicted potential distribution for the present and future. The same was found to *Ameivula nativo* and *Liolaemus lutzae*. Moreover, our results showed a potential marked reduction of potential distribution covered PAs. The recognition of potential distribution areas discussed here enables focal and urgent conservation strategies, besides bringing up alerts on protected areas’ role in conserving these species under climate change scenarios. We propose creating planning policies using space and time criteria and developing long‐term studies, besides promoting educational programs aiming at environmental conservation. Thus, we expect our research to contribute to the protection of the land over Brazilian restingas‐endemic lizards' distribution.

## Graphical abstract

We evaluated the current and future potential distribution of the endemic lizards from Brazilian restingas and hypothesize that the ENMs will indicate alterations in the potential distribution of the restinga‐endemic lizards. Our models are robust enough to explain the suitable climatic zones for the occurrence of the studied species. Which points out that the species will have their potential distribution area altered for 2081–2100.

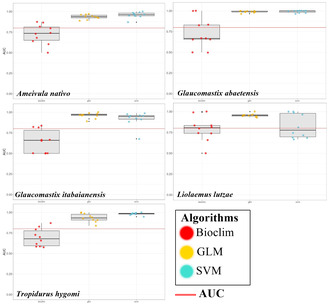

## 1 INTRODUCTION

Ecological niche models (ENMs) aim to quantify the environmental conditions suitable for species' persistence, which can be used in predicting species' current (and future) distributions (Andrade et al., 2024; Berriozabal‐Islas et al., 2017; Manes et al., 2021; Pearson et al., 2007; Srinivasulu et al., 2021). Uncovering species distributions is fundamental in assessing anthropogenic drivers that threaten species' existence and, in turn, developing optimal conservation strategies (Guisan et al., 2013; Soberón, 2007). Such is direly needed for range‐restricted habitat specialist species, as they tend to be highly sensitive to habitat loss (Burlakova et al., 2011) and climate changes (Cano Carmona et al., 2019; Zangiabadi et al., 2021).

The Atlantic Forest is a Brazilian biome considered a biodiversity hotspot due to its high endemism experiencing extensive loss of original habitat (Mittermeier et al., 2011), highlighting the urgency of its conservation. This biome exhibits high ecosystem heterogeneity, from large ombrophilous and semidecidual forests to open habitats over low‐ and high‐altitude grasslands, and mangroves (Ribeiro et al., 2011). Among the open low‐altitude habitats, the restingas are disjunct and psammophilous coastal environments dominated by herbaceous and shrubby vegetation growing on salty sandy soils by seaside (Ribeiro et al., 2011) and characterized by high solar incidence, salinity, and hydric deficit (Ormond, 1960).

The restingas of the Atlantic Forest is a key habitat home to a diverse community of lizards (Class Reptilia; Order Squamata; Suborder Sauria). About 36 species of lizards have been recorded in these restingas, of which 7 are restinga‐specialist endemics (Rocha et al., 2020). On the other hand, the anthropogenic impacts on Brazilian restingas‐endemic lizards are similar along all Brazilian coasts, such as human occupation, highways, housebuilding, or even commercial quarrying of sands (Rocha et al., 2003, 2007, 2009). Currently, only 658 ha (0.47%) of the restingas (including mangrove) ecosystem remains from the original area (Ribeiro et al., 2009). In addition, climate change raises additional concerns about these Brazilian restinga‐endemic lizards' conservation because warming climates (and rising sea levels) may make the environmental conditions in their current restinga habitats inhabitable (Diele‐Viegas & Rocha, 2018; Sinervo et al., 2010; Raz et al., 2023). More worryingly, a recent study predicted that the current spatial configuration of protected areas will be ineffective in covering environmentally suitable habitats for many species, in light of future anthropogenic climate change (Carvalho et al., 2011; Diele‐Viegas et al., 2020; Lourenço‐de‐Moraes et al., 2019). Thus, predicting the potential distribution of Brazilian restinga‐restricted species, and how this will shift in the future in light of anthropogenic climate change, is crucial for their conservation.

Here, we predicted the current and future potential distribution of the Brazilian restinga‐endemic lizards. We expected low habitat suitability of PAs in future scenarios. In this sense, we aimed to answer the following questions: (1) To what extent will the potential distribution of Brazilian restingas‐endemic lizards change under future climate change? (2) Does the current lizard distribution show a higher potential distribution than future distribution projections? (3) In the presence of potential distribution shifts, will PAs still be effective in conservation under future climate change scenarios?

## 2 MATERIALS AND METHODS

### 2.1 Studied species

**FIGURE 1:**
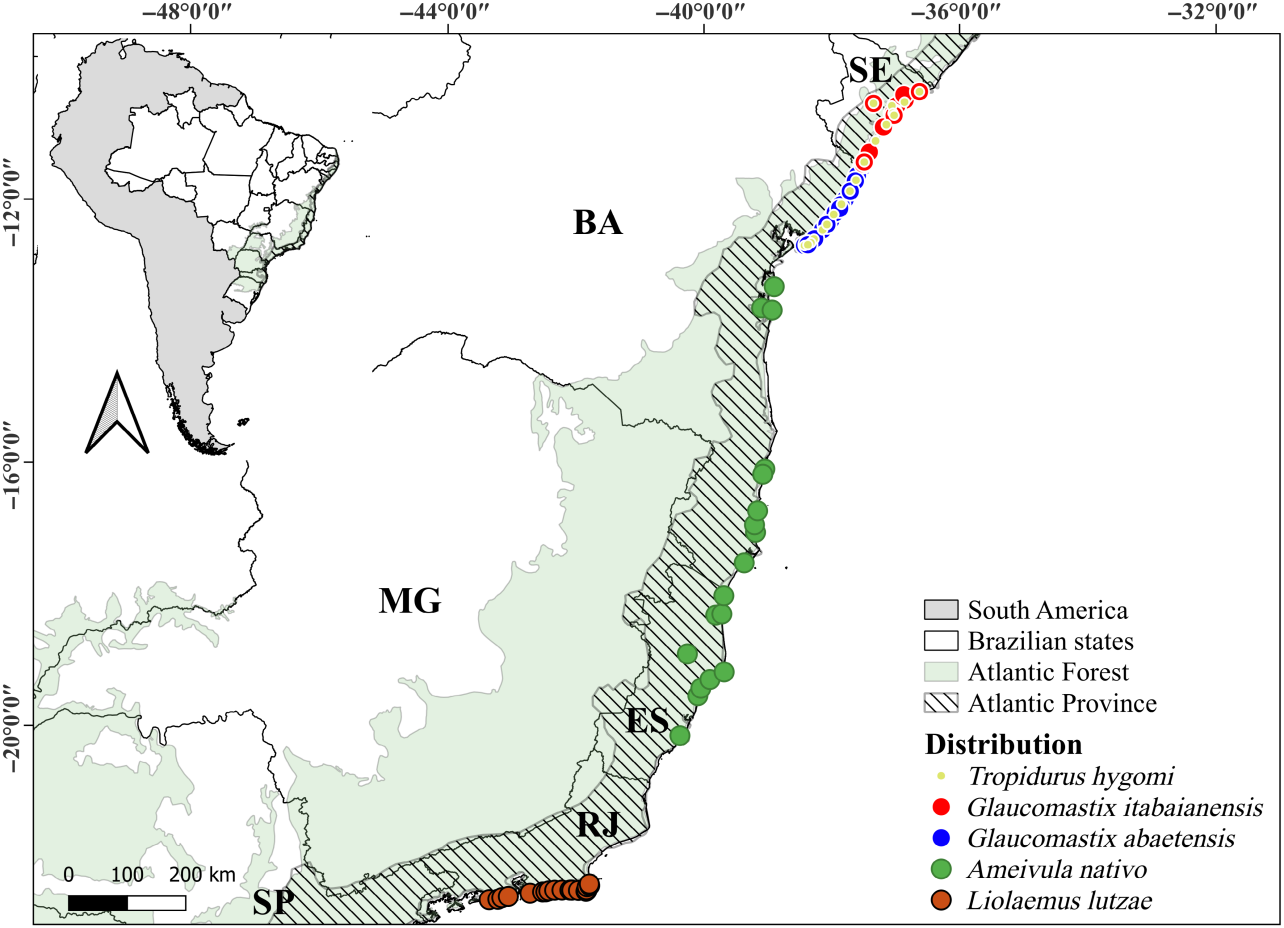
Geographic distribution of the endemic lizards from Brazilian *restingas*.

We analyzed occurrence and environmental data for five Brazilian restingas‐endemic lizards along approximately 3000 km of extension from Northern Sergipe State in northeastern southward to South Rio de Janeiro State in Southeastern Brazil (Figure 1; Table S1). Except *Glaucomastix itabaianensis* Rosário et al., 2019, a recently described Teiidae (Rosário et al., 2019), all evaluated species constitute targets of the National Action Plans (PANs) for conservation of the threatened Herpetofauna of the Brazilian Southeastern Atlantic Forest (*Herpetofauna Ameaçada da Mata Atlântica da região Sudeste do Brasil*; ICMBio, 2019a) and of the Northeastern region of Brazil (*Herpetofauna Ameaçada do Nordeste*; ICMBio, 2019b).

### 2.2 Species occurrences

We collected species occurrence records by mining published scientific literature. Then, we used the “*CoordinateCleaner*” package (Zizka et al., 2021) to filter duplicates, records within 1 km of each other, and records found in ocean. After the cleaning, 57 records were used for all sampling (Table S1): *Tropidurus hygomi* (*N* = 16), *Ameivula nativo* (*N* = 14), *Liolaemus lutzae* (*N* = 10), *G. itabaianensis* (*N* = 10), and *G. abaetensis* (*N* = 7). We did not use the restingas‐endemic lizards *Brasiliscincus caissara* (*N* = 4) and *G. littoralis* (*N* = 3) because of their low number records.

### 2.3 Environmental data

To model the species' current and future climatically suitable areas, we used the 19 bioclimatic variables available in the WorldClim 2.1 database (Fick & Hijmans, 2017). The historical data (current time) is based on monthly temperature and precipitation values from 1970 to 2000. We extracted the layers at 30‐arc‐sec (~1 km^2^) resolution and we performed a Pearson correlation analysis (Dormann et al., 2013) to identify and exclude the variables with high multicollinearity levels (≥70%) for the interest area through the “*Corrr*” package. Six bioclimatic variables were elected to proceed with the modeling: isothermality (BIO3), max temperature of warmest month (BIO5), temperature annual range (BIO7), precipitation seasonality (BIO15), precipitation of wettest quarter (BIO16), and precipitation of coldest quarter (BIO19).

For future forecasts, we used the CMIP6 climate projections provided by the WorldClim database from three global circulation models, BCC‐CSM2‐MR, MIROC6, and IPSL‐CM6A‐LR, which are considered good models for South American climatic patterns (Cannon, 2020). We considered projections for 2081–2100 based on two shared socio‐economic pathway (SSPs) scenarios: SSP3 7.0 (high CO_2_ emissions), which predicts an increase in the global average temperature of up to 4.6°C by 2100 compared to the pre‐industrial revolution period, and SSP5 8.5 (very high CO_2_ emission), for which the increase is predicted to reach up to 5.7°C (IPCC, 2021).

### 2.4 Ecological niche modeling (ENMs)

We modeled species' potential distribution using three algorithms: (1) Bioclim (Envelope; Nix, 1986); (2) generalized linear model (Statistic; McCullagh & Nelder, 1989); and (3) support vector machine (machine learning; Guo et al., 2005). Ensemble models have shown greater predictive performances than individual models by taking the consensus among the outputs of different statistical approaches (Hao et al., 2019, 2020). We chose these algorithms because their sensitivity and specificity are better applied to species with narrow distribution (Qiao et al., 2015). To avoid overprediction and low specificity, we cropped the bioclimatic layers to the Atlantic Province (sensu Morrone, 2014) in the Atlantic Forest Biome. We used the “*dismo*” package (Hijmans et al., 2023) to perform Bioclim and GLM, and the “*kernlab*” package (Karatzoglou et al., 2023) to perform the SVM models.

We sampled pseudo‐absence points across the Atlantic Province, excluding 1 Km radius buffer from occurrence records of species. We matched and randomly generated 10.000 pseudo‐absence points for GLM and 10 times the number of occurrence points for Bioclim and SVM (following Barbet‐Massin et al., 2012). Moreover, we randomly split the combined dataset (occurrence records plus pseudo‐absence data), such that 70% was used for model training and the remaining 30% for testing. We evaluated the model performance by calculating the True Skill Statistic method (TSS; Allouche et al., 2006) and the area under the curve (AUC) of receiver operator characteristics (ROC; Fielding & Bell, 1997). We generated 10 replicates for each algorithm and selected the replicates presenting accuracy values of AUC ≥0.8 and TSS ≥0.6 to construct the ensemble rasters. Then, we ensembled the models based on the average of the selected rasters weighted by their AUC values (Figure S2). All analyses were performed in R version 4.2.1 (R Core Team, 2022).

### 2.5 Climatic suitability in protected areas

Protected areas (PAs) in Brazil are categorized into two groups: integral protection (IP) and sustainable use (SU) (Brasil, 2000). The main difference between the two categories is the permission for sustainable extraction of natural resources in SU areas, which is not allowed in IPs (Vieira et al., 2019). Consequently, SU areas are, in general, comparatively more deforested than IPs (Carranza et al., 2013) and they have a more impacted fauna, as a result of human activities (Françoso et al., 2015). For this reason, we considered only IP areas to calculate the species' climatic suitability in the present and future as it is more stable for biodiversity conservation.

To calculate the climatic suitability and effectiveness of the IP areas, we considered the pixels (1 km^2^) with suitability value >0.8 for the present and future that were inside and outside PAs. The >0.8 value was determined because it is considered a reasonable limit of threshold in ecological niche models aiming at species conservation (Giannini et al., 2012; Sillero et al., 2021). All procedures were performed in R version 4.2.1 (R Core Team, 2022).

## 3 RESULTS

### 3.1 Ecological niche models (ENMs)

The ensemble models were constructed from different algorithm replicates (Bioclim, GLM, and SVM) for each species (Table S2). The Bioclim performed worst among the algorithms for all studied species (Table 1), especially those with few occurrence records or that are known to occur in only a few locations (Figure S1).

**TABLE 1:** Mean and standard deviation (±) of the evaluation metrics for each algorithm used in the ecological niche models (ENMs) of five endemic species from Brazilian restingas, Brazil.

Algorithms						
Species	Bioclim		GLM		SVM	
	AUC	TSS	AUC	TSS	AUC	TSS
*Ameivula nativo*	0.72 ± 0.12	0.45 ± 0.24	0.93 ± 0.02	0.84 ± 0.07	0.95 ± 0.03	0.91 ± 0.08
*Glaucomastix abaetensis*	0.73 ± 0.17	0.46 ± 0.35	0.99 ± 0.01	0.98 ± 0.01	0.99 ± 0.01	0.99 ± 0.01
*Glaucomastix itabaianensis*	0.64 ± 0.14	0.29 ± 0.28	0.96 ± 0.03	0.92 ± 0.07	0.91 ± 0.09	0.89 ± 0.10
*Liolaemus lutzae*	0.79 ± 0.14	0.58 ± 0.29	0.95 ± 0.02	0.87 ± 0.05	0.82 ± 0.14	0.79 ± 0.16
*Tropidurus hygomi*	0.69 ± 0.10	0.39 ± 0.20	0.93 ± 0.05	0.82 ± 0.11	0.98 ± 0.01	0.94 ± 0.03

Abbreviations: AUC, area under the curve; GLM, generalized linear model; SVM, support vector machine; TSS, true skill statistic.

**FIGURE 2:**
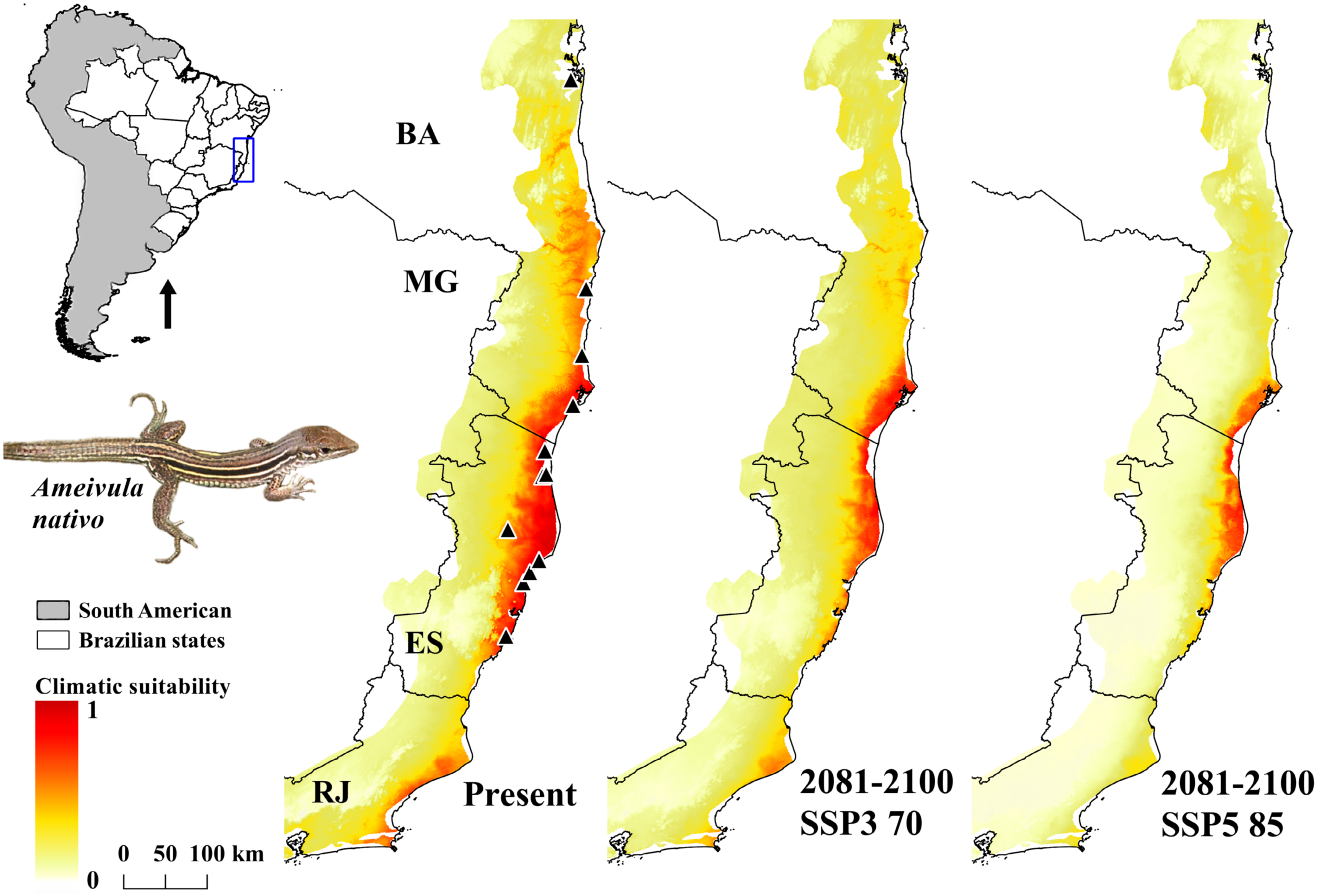
Ecological niche model (ENM) of *Ameivula nativo* in current time (present) and future (2081–2100) on two shared socio‐economic pathway (SSPs) scenarios (SSP3 70 and SSP5 85). BA, Bahia; ES, Espírito Santo; MG, Minas Gerais; RJ, Rio de Janeiro.

Most species will show substantial changes in their potential distribution area for 2081–2100 exhibiting shrinks in both climate change scenarios. The predicted distribution of *A. nativo* was consistent with the available occurrence records. This species is currently known to occur from Maraú (Bahia) southwards to Setiba (Espírito Santo) municipalities (Table S1), and only one occurrence locality in Northern Bahia did not exhibit environmental suitability (Figure 2). Moreover, the model predicts the absence of potential distribution for the species in the future, with few areas allowing its occurrence over Southeastern Bahia and the northern portion of Espírito Santo (Figure 2).

**FIGURE 3:**
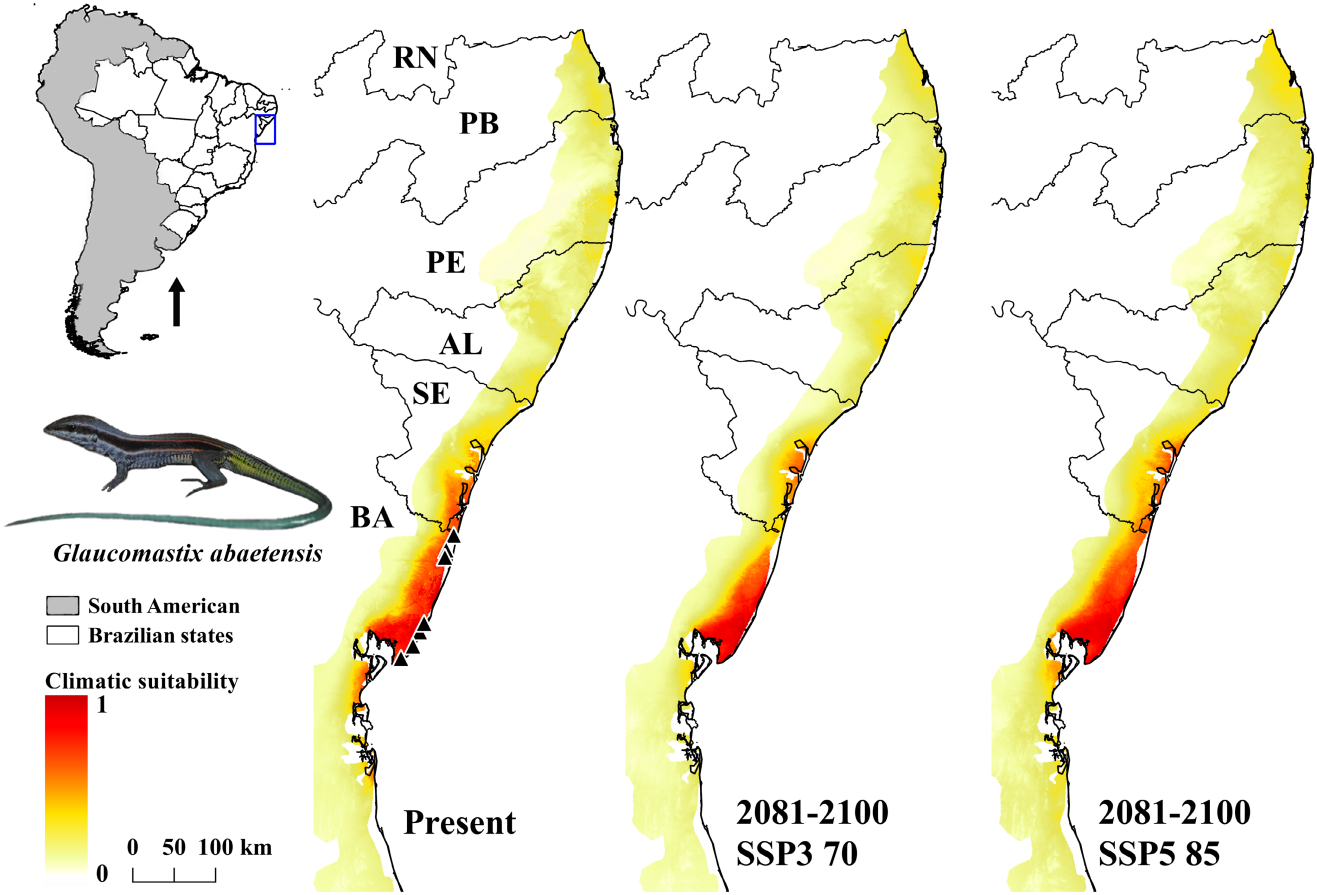
Ecological niche model (ENM) of *Glaucomastix abaetensis* in current time (present) and future (2081–2100) on two shared socio‐economic pathway (SSPs) scenarios (SSP3 70 and SSP5 85). AL, Alagoas; BA, Bahia; PB, Paraíba; PE, Pernambuco; RN, Rio Grande do Norte; SE, Sergipe.

*Glaucomastix abaetensis* had a predicted distribution similar to its current known geographic occurrence, with comparatively greater potential distribution expected for the Northern Bahia State, with less favorable conditions in the Southern Sergipe State (Figure 3). The species is currently known from Conde to Salvador municipalities in Bahia State (Table S1).

**FIGURE 4:**
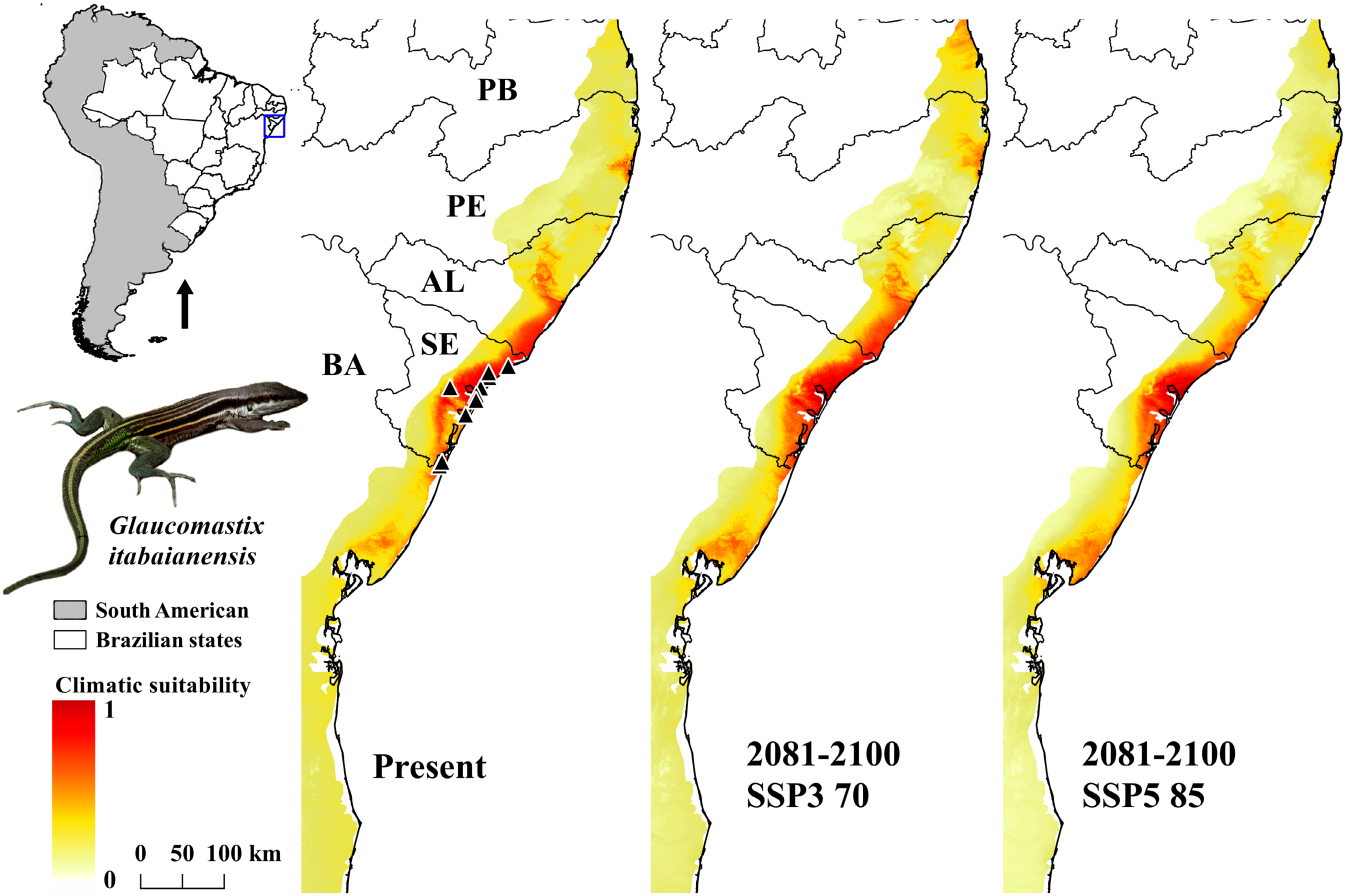
Ecological niche model (ENM) of *Glaucomastix itabaianensis* in current time (present) and future (2081–2100) on two shared socio‐economic pathway (SSPs) scenarios (SSP3 70 and SSP5 85). AL, Alagoas; BA, Bahia; PB, Paraíba; PE, Pernambuco; SE, Sergipe.

On the other hand, *G. itabaianensis* had an overpredicted distribution concerning known occurrence. Despite larger conditions in the northern portion of Sergipe State, the models recorded appropriate potential distribution in the Southern Alagoas State. Future predictions indicate restrictions in populations of the Southern Sergipe and Northern Bahia states (Figure 4). Nowadays, this species is known from Jandaíra (Bahia) to Pacatuba (Sergipe) municipalities (Table S1).

**FIGURE 5:**
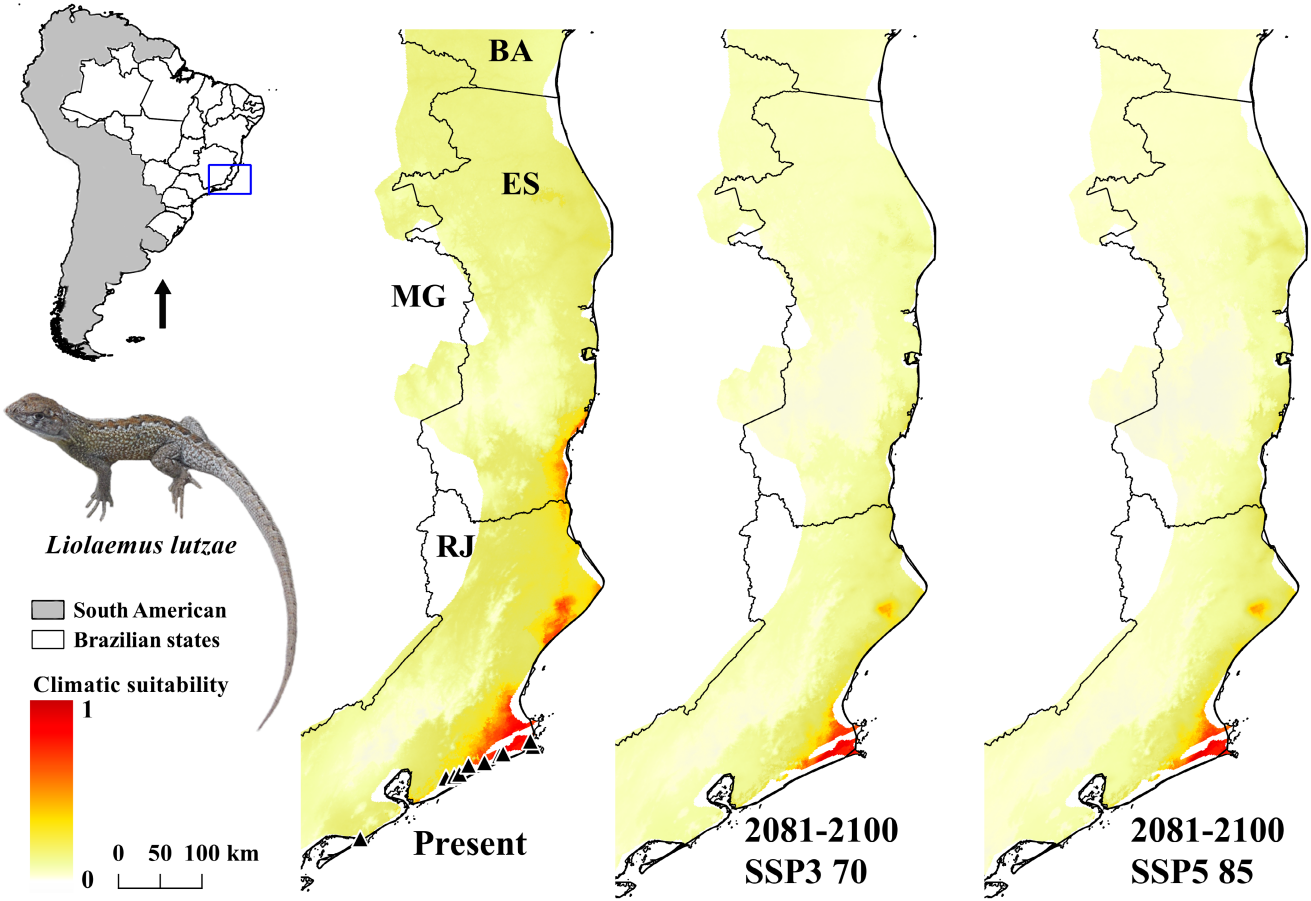
Ecological niche model (ENM) of *Liolaemus lutzae* in current time (present) and future (2081–2100) on two shared socio‐economic pathway (SSPs) scenarios (SSP3 70 and SSP5 85). BA, Bahia; ES, Espírito Santo; MG, Minas Gerais; RJ, Rio de Janeiro.

The predicted distribution of *L. lutzae* was highly consistent with the current occurrence records, except for one locality in the Southern Rio de Janeiro Municipality (Figure 5). Moreover, the current potential distribution is likely to decrease in the future. Nowadays, this species is known to occur with some remaining populations from Cabo Frio eastward to Rio de Janeiro municipalities, both situated in Rio de Janeiro State (Table S1).

**FIGURE 6:**
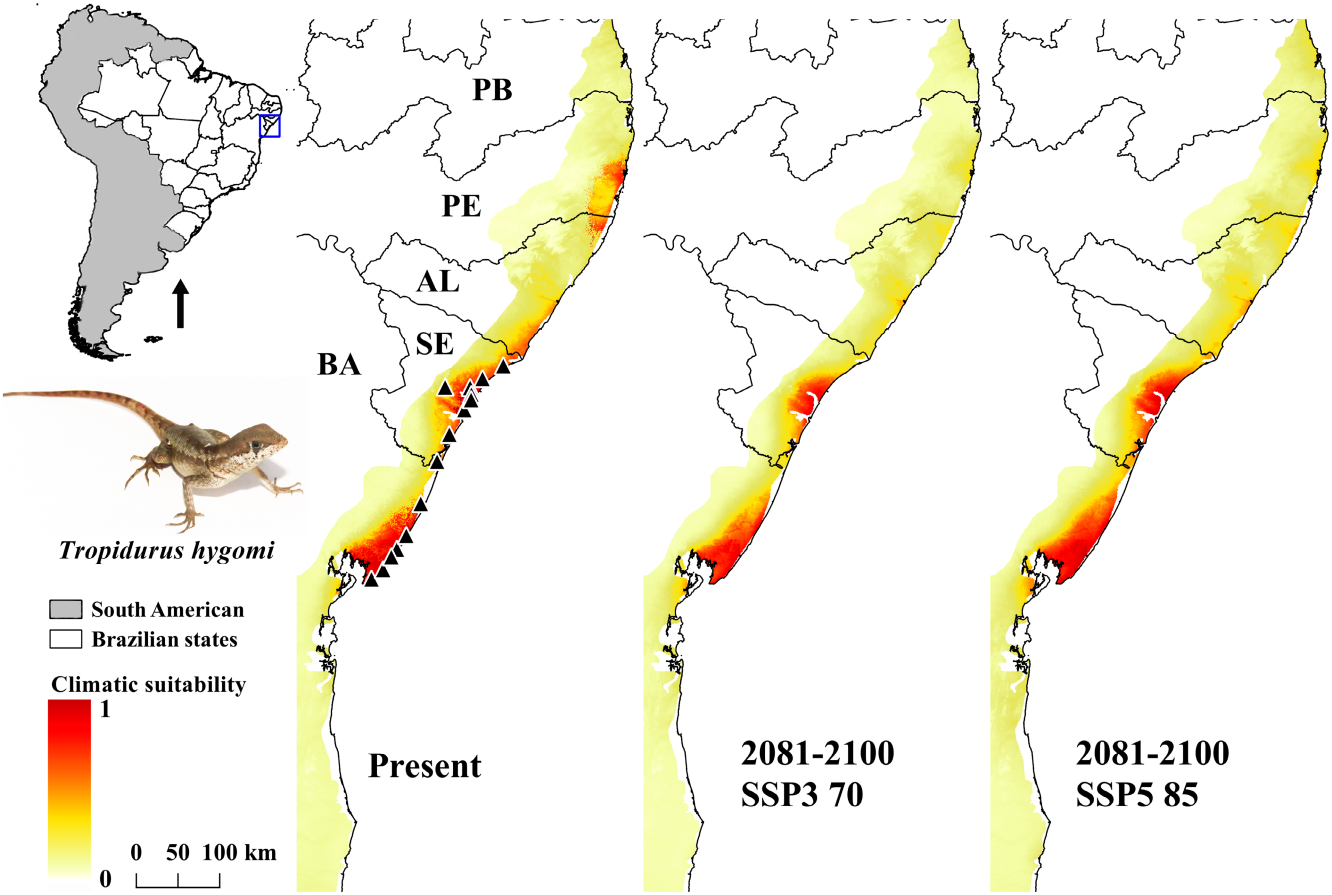
Ecological niche model (ENM) of *Tropidurus hygomi* in current time (present) and future (2081–2100) on two shared socio‐economic pathway (SSPs) scenarios (SSP3 70 and SSP5 85). AL, Alagoas; BA, Bahia; PB, Paraíba; PE, Pernambuco; SE, Sergipe.

*Tropidurus hygomi* resembled the predicted distribution compared to those we obtained for *G. abaetensis* and *G. itabaianensis*, mainly exhibiting gaps of potential distribution related to occurrence records and climatic contraction in the future with gaps in Northern Bahia and Northern Sergipe states (Figure 6). Its distribution is currently known from Salvador (Bahia) to Pacatuba (Sergipe) municipalities (Table S1).

### 3.2 Environmental suitability

Overall, the Brazilian restingas‐endemic lizards' species studied here had shrinking suitable areas, except *G. abaetensis* and *G. itabaianensis*, which presented a trend of expansion (Table 2). However, inside PAs of integral protection, the species present a contraction of their potential distribution, with some areas exhibiting zero suitable conditions for their occurrence (Table 2).

**TABLE 2:** Area (km^2^) with suitable climatic conditions for the occurrence of endemic lizard species from Brazilian restingas in 2100, considering two climate change scenarios (SSP3 70 and SSP5 85). Species exhibiting zero values did not reach 80% of climatic suitability for their occurrence. The hyphen symbol (−) refers to species without occurrence in integral protection (IP) areas.

Total distribution in km^2^				Inside of integral protection (IP) areas in km^2^		
Species	Present	Ssp3 70	Ssp5 85	Present	Ssp3 70	Ssp5 85
*Ameivula nativo*	2541,77	133,84	6486	0	0	0
*Glaucomastix abaetensis*	1294,67	1752,47	1964,53	–	–	–
*Glaucomastix itabaianensis*	1016,31	1195,09	1050,55	30,245	27,724	5879
*Liolaemus lutzae*	148,949	140,271	122,934	0	0	0
*Tropidurus hygomi*	2084,72	696,597	1923,01	0	0	0

## 4 DISCUSSION

The ENMs indicated that the species studied will experience changes in their potential distribution in the future, suggesting that the PAs will not be sufficient to protect the Brazilian restingas‐endemic lizards. The species from the northern coast of Sergipe and Bahia (*T. hygomi*, *G. abaetensis*, and *G. itabaianensis*) have some overlap along their predicted distribution for the present and future. The same relationship was found with species from the southern coast of Rio de Janeiro and Espírito Santo (*A. nativo* and *L. lutzae*).

The areas having the highest probabilities of occurrence to *T. hygomi*, *G. itabaianensis*, and *G. abaetensis* are adjusted to the current known records, except for narrow extrapolation of the model to *G. itabaianensis* in the Alagoas State and *G. abaetensis* in the Sergipe State. This result reinforces the climatic similarity of the restingas from Sergipe and Northern Bahia (Dias & Rocha, 2014; Martins et al., 2018) and Southern Alagoas states, enabling the overlap in species' potential distributions.

Previous studies on *L. lutzae* recorded areas with potential distribution in other southern regions like São Paulo, Paraná, and Santa Catarina (Winck et al., 2014). Here, our data exhibited few suitable areas besides the known records with specific climatic conditions of the Rio de Janeiro restingas. This may have resulted because the algorithm they used (MAXENT) is affected by niche breath, exhibiting sensitivity error (Qiao et al., 2015) and leading to overpredictions. Conversely, the low overprediction found here is characterized by low classification error and greater specificity attributed to the ensemble method from GLM and SVM algorithms.

Climatic changes produced refuge during the Pleistocene between the Northern Espírito Santo and Southern Bahia (Jackson, 1978), a determinant barrier delimiting species distribution (Rocha et al., 2020). Events through the penultimate (~120.000 years) and ultimate (~5.100 years) transgressions from Pleistocene (Bittencourt et al., 1983) were important to determine the current vegetation structure and the climatic conditions that allow the occurrence of endemic species along the coast (Correia et al., 2020; Dias & Rocha, 2014; Rocha et al., 2005, 2020; Rosário et al., 2019; Xavier et al., 2015). Thus, the absence of species' records in projected suitable sites can be related to the impossibility of dispersion due to geographic barriers, such as mountain chains from Serra do Mar in Southeastern São Paulo and Rio de Janeiro to *L. lutzae* (Winck et al., 2014), and rivers such as Itapicuru River to *G. abaetensis* and *G. itabaianensis* (Rosário et al., 2019), and São Francisco River between Alagoas and Sergipe states to *G. itabaianensis* and *T. hygomi* (Fazolato et al., 2017; Rosário et al., 2019).

Our models pointed to two pooled potential distributions where Brazilian restingas‐endemic lizards would potentially share these areas: (1) the Northern Coast of Bahia and Sergipe (*G. abaetensis*, *G. itabaianensis*, and *T. hygomi*); and (2) the Southeastern and Southern Coast of Bahia, Espírito Santo, and Rio de Janeiro (*A. nativo* and *L. lutzae*). Thus, we believe it is feasible that historical effects and the aforementioned ecological traits are likely to preclude species' occurrence on climatically suitable sites projected by the models. Moreover, the recognition of these potential distribution areas enables urgent and focal conservation strategies specifically aimed at them, such as establishing connections between restingas through the restoration of vegetation. Besides strategies discussed by Menezes and Rocha (2013), such as the promotion of educational programs, the establishment of periodic monitoring of populations, and examination of land use, are effective actions for conserving these areas and species.

Furthermore, our results indicated a consistent contraction in areas with potential distribution in the future as a response to climate change. Moreover, they showed an expressive contraction in suitability inside protected areas of integral protection, except for *G. abaetensis* which is not predicted to occur in any IP and did not have a predicted contraction in climatically suitable areas in the future. These results are alarming because reptiles are expected to suffer high exposure to extreme temperatures near 2100 (Murali et al., 2023). This fast and steady variation in their environmental temperatures over time can likely render them endangered in the near future (Diele‐Viegas et al., 2020). In addition, previous works highlight that current protected areas in Brazil are likely to be ineffective in achieving their goals in the future, which renders urgent careful conservation planning to evaluate areas in space and time (Carvalho et al., 2011; Lourenço‐de‐Moraes et al., 2019; Martinez‐Freiría et al., 2013). Moreover, all IPs are in areas with high anthropic pressures, such as human occupation, highways, housebuilding, or even commercial quarrying of sands (Rocha et al., 2007, 2009). Thus, we propose some alternatives to settle the problems questioned: (1) creating planning policies and management strategies for species conservation by overlapping areas with predicted environmental suitability in the present and the future; (2) creating PAs of integral protection focusing on *G. abaetensis* by using the criteria of space and time as conservation strategies; and (3) developing long‐term studies focused on these species to understand their habitat quality and population dynamics better.

Our study presents the most complete occurrence data of the endemic lizards' species from Brazilian restingas available, together with important information concerning climatically suitable areas for these species in the present and future. Moreover, these data reinforce the caution to conserve these species and present an alert about the important PAs' role in conserving these species under climate change scenarios.

## AUTHOR CONTRIBUTIONS

**Hugo Andrade:** Conceptualization (lead); data curation (lead); formal analysis (supporting); funding acquisition (equal); methodology (equal); project administration (lead); writing – original draft (lead). **Luisa Maria Diele‐Viegas Costa Silva:** Conceptualization (supporting); formal analysis (lead); methodology (supporting); supervision (equal); writing – review and editing (equal). **Carlos Frederico Duarte Rocha:** Conceptualization (equal); funding acquisition (equal); methodology (equal); writing – review and editing (equal). **Antônio Jorge Suzart Argôlo:** Methodology (equal); project administration (equal); supervision (equal); writing – review and editing (equal). **Eduardo José dos Reis Dias:** Conceptualization (supporting); methodology (supporting); project administration (supporting); supervision (lead); writing – review and editing (equal).

## CONFLICT OF INTEREST STATEMENT

The authors declare that they have no conflicts of interest.

## Supporting information


Figure S1:

Table S1:

Table S2:

Data S1:

## Data Availability

The data and species distribution that support the findings of this study are available in the Supplementary Material of this article.
