# Supplementary figures and images for: Potential distribution of endemic lizards from Brazilian restingas: The present announcing the end
Source: Ecol Evol. 2024 Nov 20;14(11):e11618. doi: 10.1002/ece3.11618 (PMC11578648; doi:10.1002/ece3.11618)

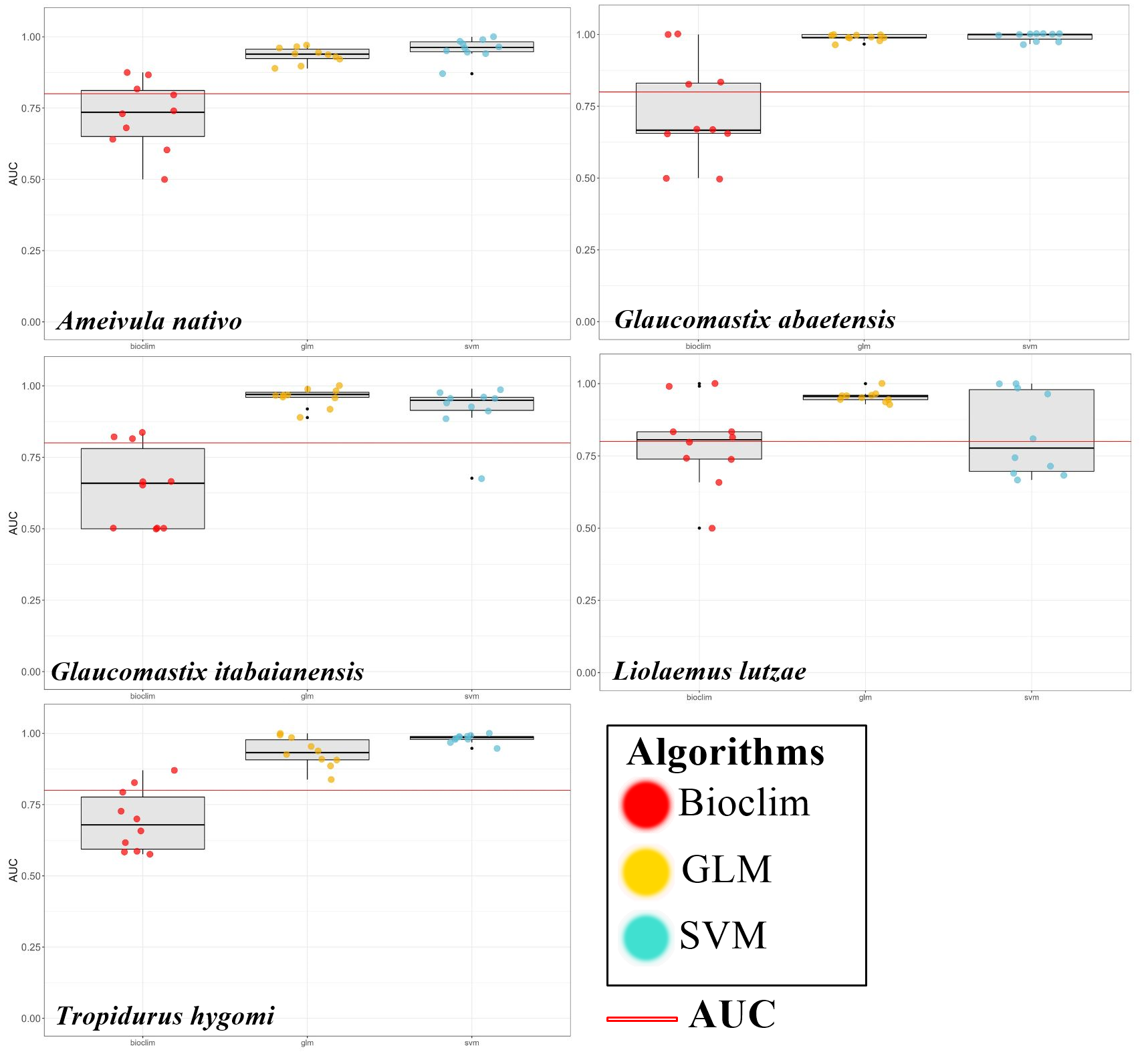

Supplement: Supplementary file 1 — Figure S1: [file ECE3-14-e11618-s001.tif]
