# Supplementary material for: Potential distribution of endemic lizards from Brazilian restingas: The present announcing the end
Source: Ecol Evol. 2024 Nov 20;14(11):e11618. doi: 10.1002/ece3.11618 (PMC11578648; doi:10.1002/ece3.11618)
Supplement: Supplementary file 4 — Data S1: [file ECE3-14-e11618-s002.docx]

Figure S1: Boxplot of the algorithm performances and models with AUC ≥0.8 were implemented on ensemble models conforming Table S2 (see Material and Methods).

Table S1: Coordinates used for species to perform the ecological niche models (ENMs). The * was used for coordinates in center of municipalities and † was used for coordinates that were excluded after utilization of the “coordinatecleaner” function (see Materials and Methods).

Table S2: Number of replicates generated from each algorithm for species, and thresholds from TSS and AUC. Only values ≥0.8 were implemented on ensemble models (see Material and Methods).
